# Impact of Different Transcatheter Edge-to-Edge Repair Systems on Tricuspid Annular Dimensions: A Three-Dimensional Imaging Analysis

**DOI:** 10.1016/j.shj.2025.100758

**Published:** 2025-11-16

**Authors:** Jennifer von Stein, Nina C. Wunderlich, Maria Isabel Körber, Stephan Baldus, Juan F. Granada, Roman Pfister, Christos Iliadis, Philipp von Stein

**Affiliations:** a University of Cologne, Faculty of Medicine and University Hospital Cologne, Department III of Internal Medicine, Cologne, Germany; b Cardiovascular Research Foundation, New York, New York, USA; c Asklepios Klinik Langen, Med. Klinik I/ Kardiologie, Angiologie, Internistische Intensivmedizin, Langen, Germany

**Keywords:** 3D transesophageal echocardiography, PASCAL, Remodeling, TriClip, Tricuspid annulus, Tricuspid regurgitation, T-TEER

## Abstract

• Both TriClip and PASCAL induced similar and significant acute tricuspid annular remodeling as assessed by three-dimensional transesophageal echocardiography.
• Device design features, including independent leaflet capture and gripper/clasp geometry, may enable different intraprocedural approaches but result in similar overall geometric effects.

Tricuspid valve (TV) transcatheter edge-to-edge repair (T-TEER) is an established therapy for severe tricuspid regurgitation (TR) in patients at prohibitive surgical risk.^1^ T-TEER effectively reduces TR, improves symptoms, quality of life, and annualized heart failure hospitalization rates.^2^ In addition to the surgical principle of leaflet approximation, T-TEER has been associated with an indirect annuloplasty effect, with smaller mid-right ventricular diameter and the Clover strategy emerging as independent predictors of tricuspid annular (TA) remodeling.^3^

However, comparative data on the two available T-TEER systems, TriClip (Abbott) and PASCAL (Edwards Lifesciences), regarding TA remodeling, are lacking. Although both T-TEER devices rely on the same principal mechanism, distinct design features may influence the extent of annuloplasty. We therefore assessed the acute impact of TriClip and PASCAL on TA dimensions using intraprocedural 3-dimensional (3D) transesophageal echocardiography in consecutive patients undergoing T-TEER at the University Hospital Cologne. The study was approved by the Institutional Review Board, and all patients provided written informed consent. All examinations were performed on a Vivid E95 system (GE Vingmed Ultrasound, Horten, Norway). To ensure balanced group sizes, we restricted inclusion to patients treated between 2020 and 2021. Device selection was at the interventionalist’s discretion, with both systems available throughout the study period. Intraprocedural preimplantation and postimplantation TA area, perimeter, septolateral, and anteroposterior diameters were manually measured offline in early diastole from 3D transesophageal echocardiography data sets using multiplanar reconstruction with dedicated software (TOMTEC Imaging Systems GmbH, Unterschleissheim, Germany) by 2 experienced echocardiographers (Figure 1). Interobserver and intraobserver agreement for annular measurements was excellent, with intraclass correlation coefficients of 0.92 to 0.95 for area and perimeter, and 0.82 to 0.92 for septolateral and anteroposterior diameters.

**Figure 1:**
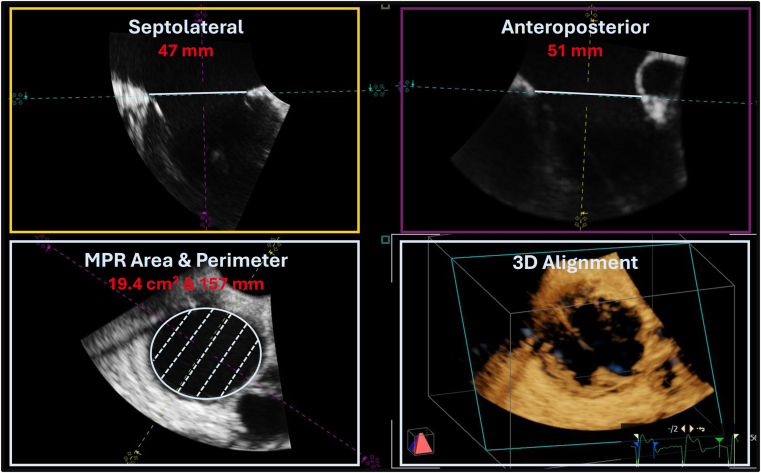
**3D TEE multiplanar reconstruction for tricuspid annular dimension assessment.** Representative example of preprocedural 3D TEE measurement of TA dimensions using multiplanar reconstruction. Orthogonal long- and short-axis planes were aligned along the TA to enable accurate tracing of its area, perimeter, septolateral, and anteroposterior diameters in early diastole. Abbreviations: 3D, 3-dimensional; MPR, multiplanar reconstruction; TA, tricuspid annular; TEE, transesophageal echocardiography.

Analysis of covariance was applied to assess baseline-adjusted changes in TA dimensions between devices. To explore potential influences of TR phenotype, patients were categorized into atrial (AFTR) and ventricular functional (VFTR) subtypes. AFTR was defined by a TA plane systolic excursion >17 ​mm and a left ventricular ejection fraction ≥50%, whereas patients not meeting one or both criteria were classified as VFTR. In addition, residual TR ​≤ ​2+ at discharge and latest follow-up were analyzed.

A total of 53 patients (74% female, median age 80 [75–83] years, New York Heart Association III/IV 87%) were included (TriClip N ​= ​23, PASCAL N ​= ​30). Most presented with atrial fibrillation (94%) and secondary TR (96%), with 34% AFTR and 66% VFTR. The distribution of subtypes was similar between devices (*p* ​> ​0.999). Patients frequently showed right heart dilation, mild right ventricular dysfunction, and severe or greater TR, with no differences between groups. Septolateral coaptation gap (4 [2–7] vs. 5 [3–6] mm; *p* ​= ​0.679), complex valve morphology (>3 leaflets; 52% vs. 63%; *p* ​= ​0.555), septal leaflet length (17.4 ​± ​3. vs. 16.8 ​± ​3.5 ​mm; *p* ​= ​0.642), and TV tenting height (10.9 ​± ​3. vs. 10.7 ​± ​3.8 ​mm; *p* ​= ​0.905) were similar between TriClip- and PASCAL-treated patients.

TriClip patients received XT or XTW, with a total of 42 devices implanted (median 2 [1–2] per patient, ≥2 devices in 57%). All PASCAL patients received the PASCAL Ace, with a total of 53 devices implanted (median 2 [2–2] per patient; ≥2 devices in 27%; *p* ​= ​0.085). The median procedure time was 135 (110–150) minutes for TriClip and 150 (117–190) minutes for PASCAL (*p* ​= ​0.069). Implant positions were anteroseptal and posteroseptal in 55%, anteroseptal in 36%, and posteroseptal in 9% (*p* ​= ​0.908 between groups), corresponding to a Clover strategy in 88% of TriClip and 73% of PASCAL cases (*p* ​= ​0.484). Independent leaflet capture was more frequently used in the PASCAL group (96 vs. 9%; *p* ​< ​0.001). Single-leaflet device attachment was rare, occurring in 1 TriClip patient and in none with PASCAL (*p* ​= ​0.893). Residual TR ​≤ ​2+ was achieved in 70% TriClip and 83% PASCAL (*p* ​= ​0.392).

Paired analysis demonstrated significant reductions in all TA dimensions in both device groups (all *p* ​< ​0.05). Analysis of covariance revealed no between-group differences (Figure 2). When adjusted for baseline annular dimensions and device type, no significant differences in TA remodeling were observed between AFTR and VFTR. At latest available follow-up (median 202 days [47–728]), residual TR ​≤ ​2+ was maintained in 73% (*N* ​= ​16/22) TriClip vs. 75% (N ​= ​21/28) PASCAL (*p* ​> ​0.999).

**Figure 2:**
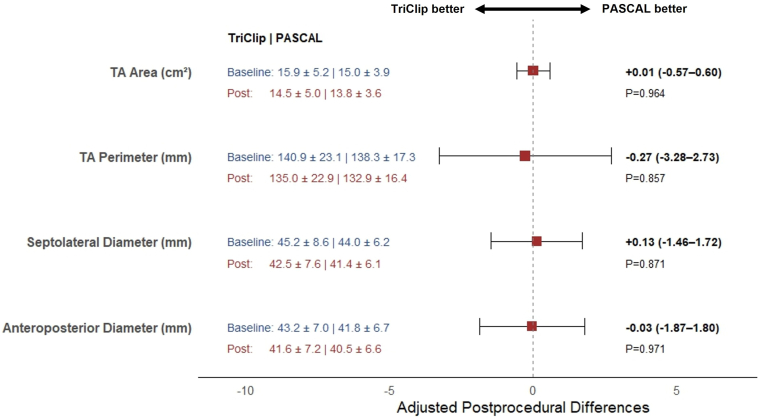
**Adjusted comparison of acute changes in tricuspid annular dimensions between devices.** Forest plot showing baseline-adjusted between-group differences (mean ± 95% CI) in postprocedural TA area, perimeter, septolateral, and anteroposterior diameters, derived from ANCOVA. Mean baseline values (blue) and postprocedural values (red) for TriClip (left) and PASCAL (right) are displayed alongside for context. The red marker represents baseline-adjusted postprocedural differences. Abbreviations: ANCOVA, analysis of covariance; TA, tricuspid annular.

Our study provides the first 3D echocardiographic evidence that both TriClip and PASCAL induce similar and significant acute TA remodeling. T-TEER is a leaflet-based therapy, exerting indirect annuloplasty by centripetal traction that approximates opposing tricuspid leaflets. Although both Conformité Européenne (CE)-marked devices share the same principal mechanism, they differ in several design features: First, the TriClip implant is composed of a rigid cobalt–chromium alloy, whereas the PASCAL device is constructed from flexible nitinol, raising the hypothesis that the more compliant PASCAL frame might attenuate indirect annuloplasty. Second, the TriClip (XT/XTW) secures the leaflets between its ∼12 ​mm long and 4–6 ​mm wide cobalt–chromium arms and the nitinol-based grippers, each containing 6 rows of friction elements and closing through an active locking mechanism. By contrast, the PASCAL Ace secures the leaflets between ∼10 ​mm long and ∼6 ​mm broad contoured paddles and a passive, spring-loaded clasp mechanism with a single row of distal hooks. The longer TriClip arms and their active locking mechanism may enable deeper leaflet insertion and more robust fixation, whereas the broader contoured paddles of PASCAL may allow for more atraumatic leaflet capture and broader leaflet traction. The extent of leaflet tissue capture may influence the degree of indirect annuloplasty, as deeper leaflet insertion could theoretically increase centripetal traction on the annulus, although this was not assessed in our study. Third, both systems permit independent leaflet capture, but this strategy was more frequently applied with PASCAL, which might have been advantageous in bridging larger coaptation gaps and optimizing device position, especially in the TV, where more than 3 leaflets are frequently encountered. Beyond these device-specific features, implantation strategies may also contribute to annuloplasty. In a previous analysis, Clover configuration, a strategy previously associated with greater annular reduction,^3^ and the use of ≥2 devices were numerically more frequent with TriClip. Importantly, residual TR at discharge and follow-up was similar between TriClip and PASCAL, underscoring the overall safety and effectiveness of both systems in achieving sustained clinical benefit.

Ultimately, these considerations may explain why device-specific design elements did not translate into measurable differences in annular remodeling in our cohort. However, this study is hypothesis-generating with important limitations, including the retrospective design, small sample size, and the nonrandomized device selection, which may have introduced selection bias. Moreover, multiplanar reconstruction may oversimplify the nonplanar TA geometry, whereas emerging semiautomated or fully automated 3D quantification techniques could allow a more accurate assessment in the future. Whether acute TA remodeling translates into durable TR reduction and improved outcomes will require further investigation in larger prospective studies.

## Ethics Statement

The study was conducted in accordance with the principles outlined in the Declaration of Helsinki. The study protocol was approved by the Institutional Review Board, and all patients provided written informed consent.

## Funding

The authors have no funding to report.

## Disclosure Statement

Jennifer von Stein has received speaker honoraria and travel expenses by Edwards Lifesciences. Christos Iliadis has received travel support by Abbott Structural and Edwards Lifesciences and consultant honoraria by Abbott Structural and Edwards Lifesciences. Stephan Baldus has received honorarium for consultation by Abbott Structural and Edwards Lifesciences. Roman Pfister has received speaker fees by Edwards Lifesciences and Abbott Structural. Maria Isabel Körber has received travel support by JenaValve and lecture fees from Edwards Lifesciences and Abbott Structural.

The other authors had no conflicts to declare.
